# Progress, pharmacokinetics and future perspectives of luteolin modulating signaling pathways to exert anticancer effects: A review

**DOI:** 10.1097/MD.0000000000039398

**Published:** 2024-08-23

**Authors:** Rui Wang, Xia Li, Yanhan Xu, Yangyang Li, Weisong Zhang, Rongqi Guo, Jianxiang Song

**Affiliations:** a Department of Thoracic Surgery, The Sixth Affiliated Hospital of Nantong University, Yancheng Third People’s Hospital, Yancheng, PR China; b Medical School of Nantong University, Nantong, PR China; c Department of General Medicine, The Sixth Affiliated Hospital of Nantong University, Yancheng Third People’s Hospital, Yancheng, PR China.

**Keywords:** anti-cancer, immunity, luteolin, NPs, pharmacokinetics, signalling pathways

## Abstract

Luteolin (3, 4, 5, 7-tetrahydroxyflavone) are natural flavonoids widely found in vegetables, fruits and herbs, with anti-tumor, anti-inflammatory and antioxidant effects, and also play an anti-cancer effect in various cancers such as lung, breast, prostate, and liver cancer, etc. Specifically, the anti-cancer mechanism includes regulation of various signaling pathways to induce apoptosis of tumor cells, inhibition of tumor cell proliferation and metastasis, anti-angiogenesis, regulation of immune function, synergistic anti-cancer drugs and regulation of reactive oxygen species levels of tumor cells. Specific anti-cancer mechanisms include regulation of various signaling pathways to induce apoptosis, inhibition of tumor cell proliferation and metastasis, anti-angiogenesis, reversal of epithelial-mesenchymal transition, regulation of immune function, synergism with anti-cancer drugs and regulation of reactive oxygen species levels in tumor cells. This paper integrates the latest cutting-edge research on luteolin and combines it with the prospect of future clinical applications, aiming to explore the mechanism of luteolin exerting different anticancer effects through the regulation of different signaling pathways, so as to provide a practical theoretical basis for the use of luteolin in clinical treatment and hopefully provide some reference for the future research direction of luteolin.

## 1. Introduction

Malignant tumors, as a multi-causal, systemic and complex disease, are characterized by uncontrolled cell proliferation and distant metastasis of cancer cells. According to statistics released by the World Health Organization (WHO), in 2020, China will have 4.57 million new cancer cases, accounting for 23.7% of the world total, and 3 million deaths, accounting for 30% of the world total. The number of new cases and deaths ranked first in the world, indicating that the burden of malignant tumors is significantly higher in China, and some studies have found that cancer has become the leading cause of death among Chinese urban and rural residents, and the incidence rate is increasing year by year.^[1]^ Despite continuous innovation in cancer treatment and diagnostic tools in recent years, the overall survival rate of cancer patients is still low, which may be related to the fact that most patients have already developed distant metastases at the time of diagnosis,^[2]^ and although chemotherapy and targeted therapy are used as the main treatment for advanced malignant tumors, drug toxicity, side effects and drug resistance during treatment often make the therapeutic effect less than optimal. Therefore, there is an urgent need for more effective, safer and more sensitive drugs in the comprehensive treatment of malignant tumors.

The plant kingdom is a major source of natural compounds that have been used for centuries as powerful tools in the treatment of advanced malignancies due to their excellent drug safety and desirable efficacy. Some studies have confirmed that flavonoids from the plant kingdom have good anticancer properties in both cells and animal models.^[3]^ And luteolin are one of the most widely studied flavonoids.

This review focuses on integrating the cutting-edge research on luteolin, highlighting the new discoveries of luteolin in regulating signaling pathways and synthesizing the high-level research in recent years, which makes this paper more novel in terms of prospective information. Moreover, this paper elaborates the detailed mechanisms of luteolin in anticancer signaling pathways, especially involving new signaling pathways and molecular interactions, and the in-depth analysis of these mechanisms can help to reveal the new dimensions of anticancer effects of luteolin, and this paper also highlights the prospects of luteolin in clinical applications, which increases their practicality and novelty.

## 2. Source of luteolin and characterization of their chemical structure and biosynthesis

Lignan (3′,4′,5,7-tetrahydroxyflavone) is a naturally occurring flavonoid, so called because it was originally isolated from the herb lignan, genus lignan in the family Lignaceae,^[4]^ and belongs to a class of phytoestrogens. It is mainly found in the form of glycosides in vegetables, fruits and herbs, such as carrots, broccoli, onions, chilli peppers, apples, oranges, honeysuckle, chrysanthemums and Salvia divinorum, etc.^[5]^ There are two main types of glycosylation of glycoside elements: one is through the free hydroxyl (OH) group, called O-glycoside, and the other is through the C-C bond, called C-glycoside.

**Figure 1.**
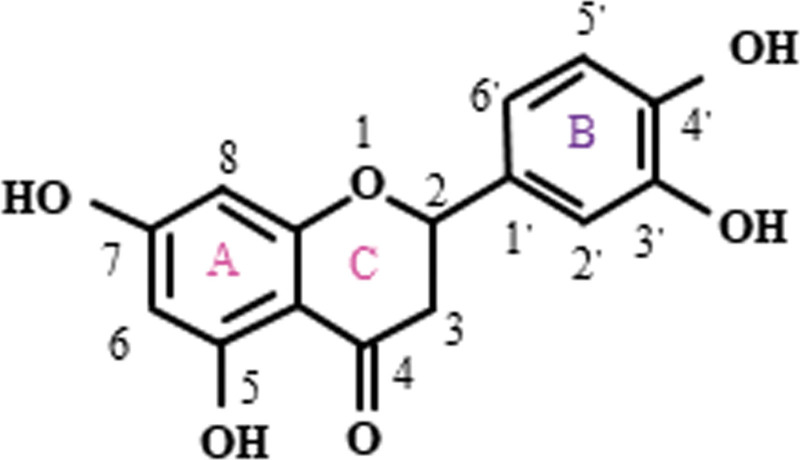
Structure of luteolin.

Luteolin is a yellow acicular body containing water of crystallisation, its molecular formula is C15H10O6 and its chemical structure is shown in (Fig. 1), the basic structure is C6-C3-C6, it contains two benzene rings and an oxygenated ring containing a carbon-carbon double bond.^[6]^ From the molecular structure, it is found that it is a polyphenol hydroxyl compound, and due to the existence of intermolecular forces between the hydroxyl groups, its lipophilicity and hydrophilicity are poor, but of course, it is also due to the presence of hydroxyl parts on the 5, 7, 3′ and 4′ carbons in the structure of luteolin and the existence of 2 to 3 double bonds, which determines that luteolin has a variety of pharmacological effects.^[7]^

**Figure 2.**
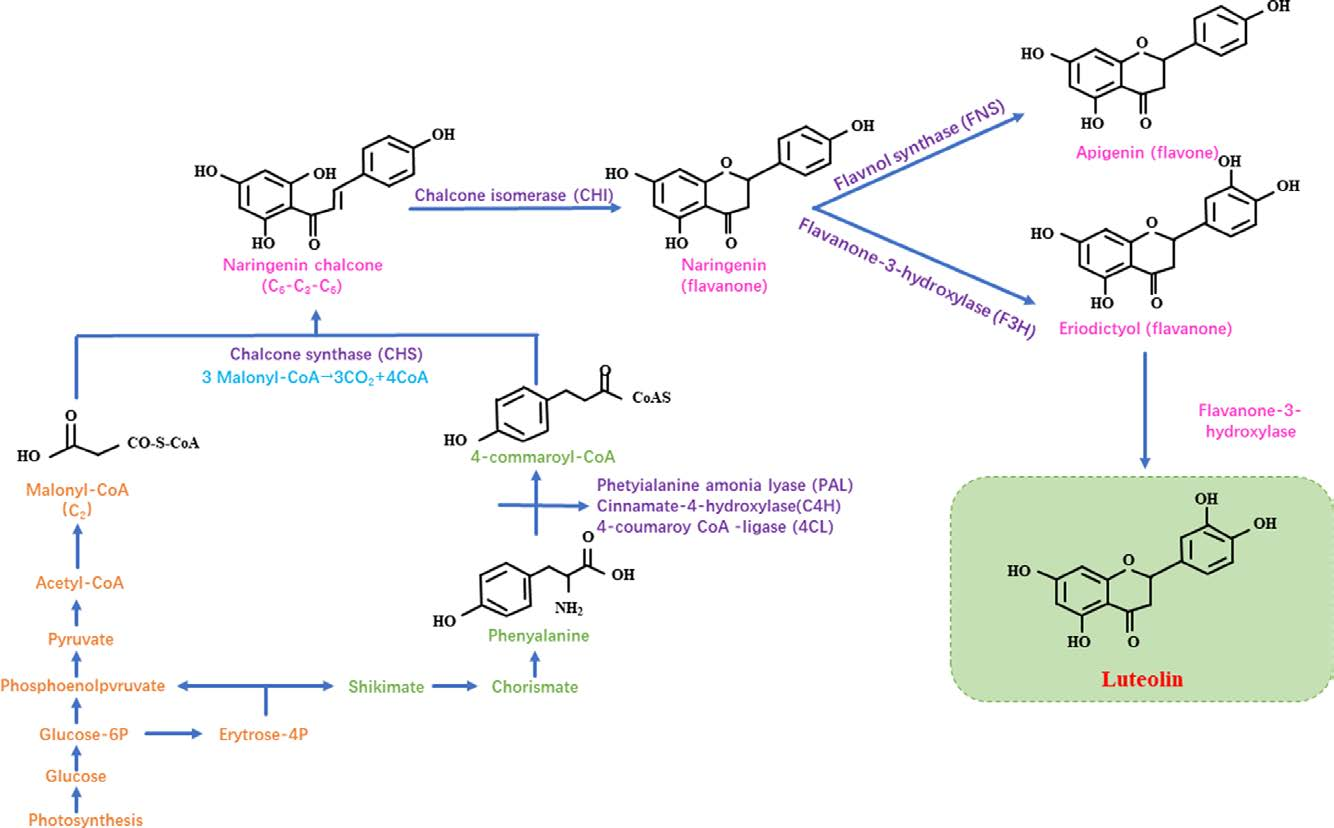
Biosynthesis of luteolin.

Luteolin are synthesized by condensation reactions involving several enzymes and their synthesis requires the phenylalanine pathway. First, L-phenylalanine is converted in three steps by phenylalanine deaminase, cinnamate 4-hydroxylase and 4-coumarol coenzyme A ligase to 4-coumarol coenzyme A, a common intermediate for flavonoids.^[7,8]^ Chalcone synthase then condenses a 4-coumarol coenzyme A molecule with three propylene glycol coenzyme A molecules to form naringenin chalcone, which is the backbone of all flavonoids.^[8,9]^ Heterocycle C is then catalyzed by chalcone isomerase (CHI) to produce naringenin, the precursor of luteolin and apigenins. Finally, in the presence of flavonoid 3′-hydroxylase, apigenin continues to serve as a substrate for the production of luteolin (Fig. 2).

## 3. Physiological and pharmacological effects of luteolin

Luteolin have been shown to have a variety of physiological and pharmacological effects, such as antioxidant,^[10]^ anti-aging,^[11]^ modulation of inflammation-related pathways thereby reducing inflammation,^[12–14]^ attenuation of atherosclerosis^[15,16]^ and amelioration of myocardial ischemia-reperfusion injury.^[17]^ In addition, luteolin are also natural anticancer agents, and a large number of studies have shown that they can significantly inhibit the development of cancers such as melanoma,^[18]^ breast cancer,^[19]^ bladder cancer,^[20]^ non-small cell lung cancer,^[21]^ laryngeal cancer,^[22]^ and gastrointestinal malignancies,^[23–25]^ etc. It is worth noting that the anti-inflammatory and antioxidant effects of luteolin may be correlated with their anti-cancer effects. This may be due to the fact that luteolin, as a substance with multiple biological effects, are mostly interrelated in their pharmacological actions and functions.^[26]^

### 3.1. Role of antioxidant activity of luteolin

The main reason for the multiple biological activities of luteolin is their antioxidant capacity, which has been shown to have a strong free radical scavenging ability. It can react with free radicals, providing hydrogen to the free radicals and transforming itself into stable phenoxyl radicals, thus reducing the oxidation of free radicals and preventing them from causing damage to cells.^[27]^ It can be seen that the stronger antioxidant property of luteolin can effectively scavenge free radicals during cancer development, prevent cellular DNA damage and mutation, and curb the development of cancer.

### 3.2. Anti-inflammatory effects of luteolin

Luteolin are known for their natural anti-inflammatory properties, which can modulate and inhibit the inflammatory response at multiple levels by interfering with several inflammatory pathways and modulators. Their anti-inflammatory effects have also been linked to cancer. Luteolin not only directly affect the production of certain inflammatory signals, but also reduce the release of inflammatory mediators. It inhibits the synthesis and activity of COX-2, a key enzyme in prostaglandins. At the same time, luteolin can also inhibit the activation of nuclear factor Kappa-light-chain-enhancer of activated B cells (NF-κB), a key transcription factor in the inflammatory process, thereby reducing the expression of inflammation-related genes.^[28]^ In conclusion, the modulation of inflammation-related molecules, pathways and enzymes by luteolin highlights their broad anti-inflammatory capabilities.

### 3.3. Anti-tumor effects of luteolin

In cancer development, luteolin can reverse epithelial-mesenchymal transition (EMT) by inducing the expression of the epithelial biomarker E-cadherin and downregulating the expression of the mesenchymal biomarkers N-cadherin, snail and vimentin, and also inhibit cell transformation, invasion and metastasis, and angiogenesis during cancer through various pathways such as cell cycle regulation, It can also inhibit cell transformation, invasion and metastasis, and angiogenesis during cancer by regulating the cell cycle, inducing apoptosis, modulating the level of reactive oxygen species (ROS) in tumor cells, and reducing transcription factors.^[29]^ In recent years, a great deal of research has been conducted at home and abroad on the anti-tumor mechanism of luteolin, and luteolin has been shown to induce and enhance apoptosis of cancer cells, but the specific mechanism of killing cancer cells has not been well elucidated, and a clear understanding of how luteolin plays a role in affecting the signaling pathways will better promote the role of luteolin in cancer prevention and treatment. Therefore, this paper provides a detailed review of luteolin that affect different signaling pathways and thus exert different anti-tumor effects, with the aim of providing a practical theoretical basis for the subsequent clinical use of luteolin.

## 4. Luteolin influences signaling pathways to exert antimalignant effects

A signaling pathway is a series of enzyme-catalyzed reactions that are capable of transmitting molecular signals from outside the cell into the cell, thereby triggering specific biological effects. These extracellular molecular signals, often called ligands, include hormones, growth factors, cytokines, neurotransmitters and other small molecules. Intracellular biochemical pathways are composed of different proteins that perform different physiological and biochemical functions. Within each pathway, upstream proteins are responsible for regulating the activity of downstream proteins, and this regulation usually involves either activating or inhibiting actions that alter the conformation of downstream proteins by adding or removing phosphate groups.

Key components of the signaling pathway include protein kinases and phosphatases, which can rapidly alter the conformation of downstream proteins to transduce signals. Once a ligand binds specifically to a cell membrane or intracellular receptor, a comprehensive biological response occurs. This is not just a signaling process but, more importantly, a process of gradual amplification of external signals.

**Figure 3.**
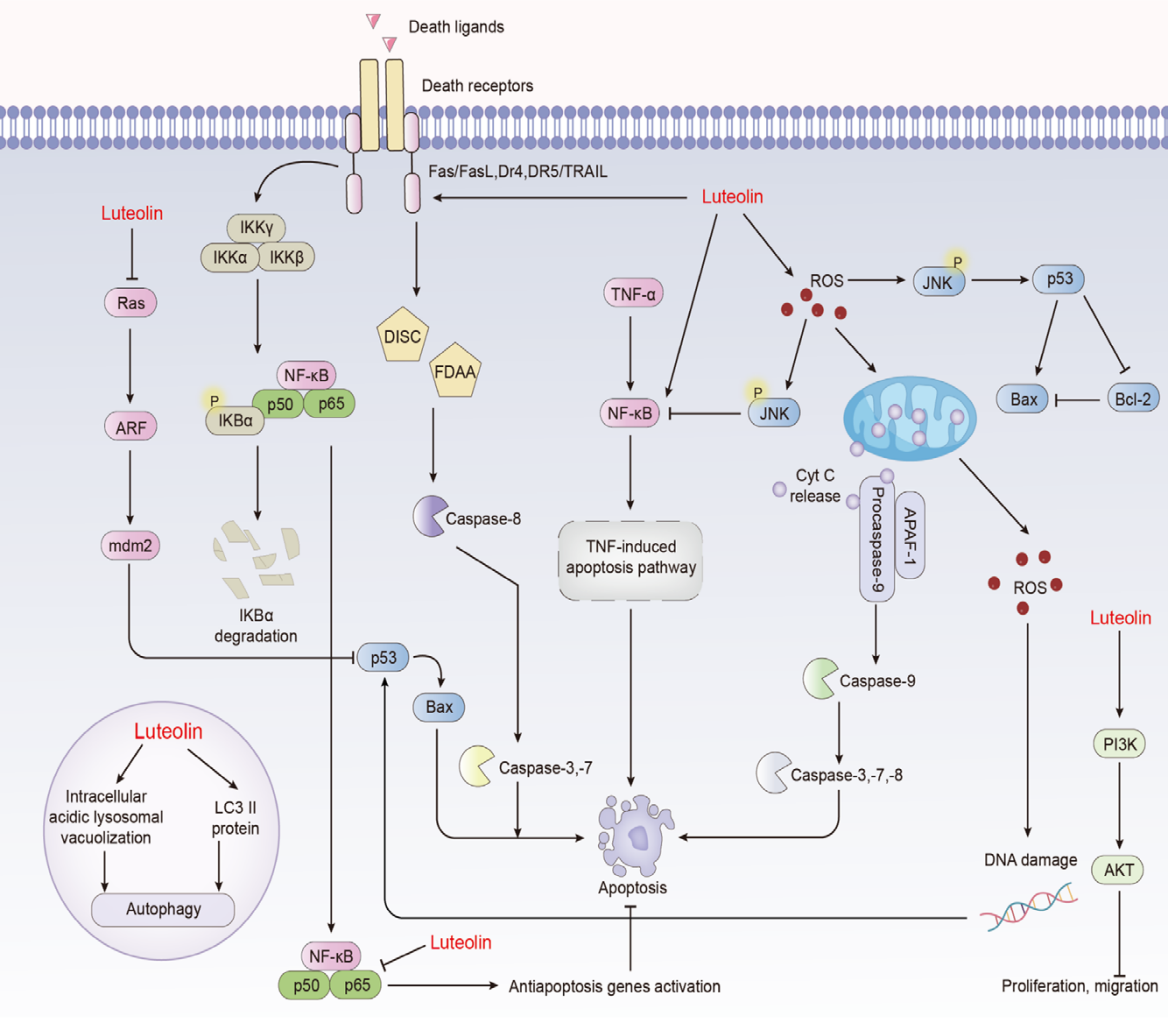
Related signaling pathways regulated by luteolin.

Receptor proteins are responsible for translating extracellular signals into intracellular signals that are subsequently amplified, propagated and modulated by signaling cascades, ultimately leading to a comprehensive set of cellular responses. These responses include regulation of downstream gene expression, alteration of intracellular enzyme activities, changes in cytoskeletal structure and adjustments in DNA synthesis. Importantly, these changes can be induced not only by single signaling pathways, but also by different combinations of different signaling pathways to produce different effects. Currently, common signaling pathways include the NF-κB pathway, phosphatidylinositol 3-kinase (PI3K)/AKT pathway, Mitogen-activated protein kinase (MAPK) pathway, Janus kinase/signal transducer and activator of transcription (JAK/STAT) pathway, TGFβ/SMAD pathway, Wnt/β-catenin pathway, Notch pathway, Hippo signaling and Hedgehog signaling. And luteolin can exert various anti-tumor effects by regulating these signaling pathways (Fig. 3).

### 4.1. Luteolin modulate the PI3K/AKT signaling pathway to exert anti-cancer effects

The PI3K/protein kinase B (PKB/AKT) signaling pathway is one of the key signaling pathways that regulate cell growth, modulate the cell cycle, influence angiogenesis and promote proliferation and metabolism, and aberrant activation of this pathway is closely associated with the development of almost all tumors and is the most commonly altered signaling pathway in human cancers.^[30,31]^

It has been shown that luteolin can inhibit the invasion and migration of non-small cell lung cancer vascular endothelial cells (NSCLC-VECs) through the miR-133a-3p/purine-rich element-binding protein B (PURB)-mediated PI3K/AKT pathway and the MAPK pathway.^[32]^ Another study also demonstrated in vitro that luteolin modulate the PI3K/AKT pathway and thus inhibit the proliferation of ovarian cancer (OC) cells and promote the apoptosis of OC cells.^[33]^ In addition, according to Eleni Bagli et al, Luteolin inhibited vascular endothelial growth factor (VEGF)-induced AKT activation in human umbilical vein endothelial cells (HUVECs), which is a downstream target of PI3K, suggesting that luteolin act on VEGF to indirectly modulate PI3K/AKT to exert an anti-angiogenic inhibitory effect on tumor proliferation.^[34]^ In a later study, Poyil Pratheeshkumar et al found in ex vivo experiments that luteolin could target the vascular endothelial growth factor receptor 2 (VEGFR2) in prostate cancer, thereby indirectly modulating the AKT/ERK/mammalian target of rapamycin (MTOR)/p70S6K/MMPs pathway, which in turn exerted the same anti-tumor effects as above.^[35]^ Other studies have shown that angiotensin II (Ang II) induces the proliferation and migration of HUVECs, but luteolin can regulate the PI3K/AKT signaling pathway to inhibit this phenomenon.^[36]^ Similarly, in a study conducted by

Xin Yao et al, it was discovered that luteolin significantly decreased the expression of matrix metalloproteinase-2 (MMP-2) and matrix metalloproteinase-9 (MMP-9) whilst also inhibiting the phosphorylation of AKT and PI3K through immunofluorescence and immunoblotting experiments. This indicates that luteolin predominantly act upon the PI3K/AKT signaling pathway, leading to the suppression of MMP-2 and MMP-9 expression. Consequently, this hinders the aggressive nature of melanoma cells and prompts their programmed cell death.^[37]^ Furthermore, luteolin up-regulate MLL3, an epigenetic regulator and tumor suppressor that is commonly altered in human cancers, inhibiting the Ras gene and subsequently inactivating the PI3K/AKT/mTOR pathway. This process ultimately results in apoptosis in drug-resistant breast cancer cells.^[38]^ In addition, some researchers have found that luteolin in combination with lapatinib, a tyrosine kinase inhibitor/targeted drug, was able to reduce the phosphorylation of AKT to exert an anti-tumor effect in breast cancer cells, suggesting that this synergistic effect is dependent on the inactivation of the AKT signaling pathway.^[39]^ This similar drug synergy was also found in another separate study, except that the synergistic drug was different, in the study by Ye-Won Jeon et al the synergistic drug taken was celecoxib (a selective cyclo-oxygenase inhibitor/non-steroidal anti-inflammatory drug) but the mechanism of antitumour was the same.^[40]^ In a study of human choriocarcinoma, it was found that Luteolin have similar synergistic effects with chemotherapeutic agents, and that luteolin can exert anti-tumor effects by inhibiting the PI3K/AKT/mTOR/SREBP cascade.^[41]^ In 2013, Kun-Chieh Chen et al discovered that luteolin could intervene in the transforming growth factor-β1 (TGF-β1)-induced PI3K/AKT/NF-κB-Snail-E-cadherin pathway, resulting in the reversal of EMT and the exertion of anti-tumor effects on lung cancer cells.^[42]^ The FOXO3a signaling pathway is a branch of the PI3K/AKT signaling pathway and is an important regulator of cell proliferation.^[43]^ In a study by Chia-Hung Lin et al, analysis of the luteolin induced signaling pathway by fluorescence quantitative PCR and protein immunoblotting showed that luteolin increased the activity of FOXO3a by inhibiting the activation of the PI3K/AKT pathway, which further induced cell cycle arrest and apoptosis in breast cancer.^[44]^ Of course, the FOXO3a pathway is not the only pathway that induces cell cycle arrest and apoptosis, luteolin can also inhibit the phosphorylation of AKT and Glycogen Synthase Kinase-3β (GSK-3β), and the activated GSK-3β can target cyclin D1 to play a role in inducing cell cycle arrest and apoptosis in nasopharyngeal carcinoma cells.^[45]^

It has been shown that insulin-like growth factor 2 (IGF-2) and insulin growth factor receptor 1 (IGF-1R) were significantly reduced in human colon cancer cells (HT-29) treated with luteolin, thereby inhibiting the activation of the IGF-1R signaling pathway and ultimately further inhibiting the PI3K/AKT pathway from exerting anti-tumor effects.^[46]^ It is noteworthy that the IGF signaling pathway can synergize with estrogen receptor (ER)-positive tumors to proliferate and metastasize,^[47]^ and many breast cancers are stimulated to progress by IGF-1. Based on this background, Wang et al suggested that ERα might act as one of the anti-tumor proliferation mechanisms of luteolin, so they interfered with the expression of ERα in human breast cancer cells (MCF-7) and found that the inhibitory effect of luteolin on IGF-1-induced cell proliferation was decreased, demonstrating that luteolin targets ERα followed by inhibition of the IGF-1-mediated PI3K/AKT pathway to exert anti-tumor effects.^[48]^ In addition, studies have reported that luteolin are not only effective in inhibiting IGF-1R, but also inhibit Akt signaling, thereby reducing the phosphorylation levels of its downstream targets, including p70S6K1, GSK-3β, and FOXO1/FOXO3.^[44,45,49]^ The specific mechanism may be that luteolin competitively bind to ATP to inhibit PI3K activity, thereby attenuating AKT phosphorylation.^[50]^

In recent years, the anti-tumor effects of luteolin via the PI3K/AKT pathway have been well studied in a considerable number of tumor cells and animal models, including nasopharyngeal carcinoma cells,^[45,51]^ choroidal melanoma cells,^[52]^ chronic myeloid leukaemia cells,^[53]^ cervical cancer cells,^[54,55]^ lung cancer cells,^[42,56]^ retinoblastoma,^[57]^ glioblastoma,^[58]^ osteosarcoma,^[59]^ papillary thyroid cancer cells,^[60]^ human tongue squamous carcinoma cells,^[61]^ esophageal squamous carcinoma cells,^[62]^ and colon cancer cells.^[63]^

In conclusion, the PI3K/AKT pathway is the main pathway through which luteolin exert their anti-tumor effects and has been extensively studied. Due to the wide variety of substrates downstream of the PI3K/AKT pathway and the different types of tumor cells, the anti-tumor effects of luteolin on the PI3K/AKT pathway also vary, but the majority of them are inhibition of cell proliferation and induction of cell apoptosis. As the pathway most prone to abnormal activation in cancer cells, the PI3K/AKT pathway remains the most important area to investigate the mechanism of the anti-tumor effect of luteolin.

### 4.2. Luteolin modulate the MAPK signaling pathway to exert anti-cancer effects

MAPK, a member of the serine (Ser)-threonine kinase (Thr) family, is an important signaling pathway that converts extracellular stimuli into intracellular responses, and activation of this pathway leads to the phosphorylation of related substrates involved in cell proliferation, apoptosis, growth and inflammation. The MAPK pathway can be divided into three distinct subpathways according to the subtypes of MAPK: c-Jun N-terminal kinase (JNK), p38 MAPK and extracellular signal-regulated protein kinase (ERK). The MAPK signaling pathway is divided into 3 different sub-pathway depending on the MAPK isoforms: the JNK signaling pathway, the p38 MAPK signaling pathway and the extracellular signal-regulated protein kinase (ERK) signaling pathway, which exert different biological effects and are closely associated with many tumourigenesis and development processes.^[64]^ Many studies have found that luteolin can exert various biological effects by regulating the MAPK pathway, including anti-inflammatory,^[65]^ anti-aging,^[66]^ promoting bone remodeling,^[67]^ and attenuating myocardial ischemia-reperfusion injury.^[68]^ As this review focuses on the anticancer effects of MAPK modulation by luteolin, other biological effects will not be described in detail.

In a study by Lu et al, Luteolin were found to induce apoptosis in gastric cancer cells (BGC-823) by upregulating the mRNA levels of dual-specific phosphatases (DUSPs), thereby inhibiting the protein phosphorylation of ERK1/2. In addition, this study also found that luteolin reduced the mRNA levels of CXC chemokine ligand 16 (CXCL16) to inhibit the PI3K/AKT pathway to exert the same anticancer effect.^[69]^ Similarly, in another study, scholars detected the expression levels of P-ERK, ERK, P-JNK, JNK, p38, P-p38, Bax (a pro-apoptotic factor), and Bcl-2 (an anti-apoptotic factor) in glioma cells (LN38) after luteolin acid treatment, and found that the expression of P-ERK, P-JNK, P-p38, and Bax was significantly up-regulated, while the expression of ERK, JNK, and p38 was unchanged, and the expression of Bcl-2 was significantly reduced, indicating that luteolin acid activated the MAPK pathway to induce apoptosis in glioma cells. were significantly up-regulated, whereas the expression of ERK, JNK, and p38 remained unchanged, and the expression of Bcl-2 was significantly reduced, suggesting that luteolin activates the MAPK pathway to induce apoptosis in glioma cells.^[70]^ Glioma cells contain astrocytomas, and in Che et al it was found that luteolin attenuated astrocyte activation and interleukin 33 (IL-33) production by interfering with the MAPK, NF-κB, and STAT3 signaling pathways.^[71]^ Luteolin induces apoptosis in human colon cancer cells not only by targeting the PI3K/AKT pathway,^[63]^ but according to Kang et al^[72]^ the JNK and p38 MAPK pathways are also involved in luteolin-induced apoptosis in human colon cancer cells. Moreover, in a more recent study in 2022, Song et al found that luteolin could inhibit cell proliferation, block the cell cycle, and induce DNA damage and apoptosis in colorectal cancer cells by inhibiting the MAPK pathway based on bioinformatics analysis in conjunction with related experiments, and it is worth noting that this study also demonstrated that combination with smetinib (a selective MEK1/2 inhibitor) significantly enhanced this effect, and combination with cisplatin significantly reduced the survival of colorectal cancer cells.^[73]^ According to Cai et al,^[74]^ Luteolin were found to inhibit tumor necrosis factor α (TNF-α)-mediated NF-κB (p65) translocation by activating JNK, thereby contributing to apoptosis in non-small cell lung cancer cells, while in a separate study, Luteolin were also found to activate the mitochondrial apoptotic pathway by phosphorylating JNK and inhibiting NF-κB translocation, ultimately inducing the apoptotic effects of A549.^[75]^ Other studies targeting lung cancer have found that luteolin causes an increase in the accumulation of superoxide, which activates JNK, which in turn triggers the rapid degradation of mitogen-activated protein kinase-1 (MKP-1) and ultimately the death of lung cancer cells.^[76]^ In hepatocellular carcinoma cells, Luteolin were found to inhibit hepatocyte growth factor (HGF)-induced cytoskeletal changes, and luteolin were confirmed to inhibit the phosphorylation of HGF membrane receptors c-Met, ERK1/2 and AKT, suggesting that the effect of Luteolin in inhibiting HGF-induced invasion of hepatocellular carcinoma cells is mediated through the MAPK/ERKs and PI3K/AKT signaling pathways.^[77]^ In CNE1 nasopharyngeal carcinoma cells, Chen et al found that Luteolin could inhibit nasopharyngeal carcinoma cell growth and motility and reduce nasopharyngeal carcinoma stem cell properties by inhibiting ERK1/2 and p38 MAPK activities.^[78]^ In addition, Luteolin have been reported to have the potential to inhibit MAPK and p-ERK1/2 activation in epidermal growth factor (EGF)-induced MCF-7 breast cancer cells.^[5]^

The regulation of MAPK and its three sub-pathway by Luteolin not only exerts anti-tumor effects, but has also been widely demonstrated in other aspects, for example, Luteolin are able to alleviate glomerular basement membrane hyperplasia caused by chronic renal failure by inhibiting the JNK/MAPK pathway.^[79]^ Luteolin can improve neutrophilic asthma by inhibiting IL-36γ activation of the MAPK pathway.^[80]^ It was also found that the reduction of phosphorylation levels of p38 MAPK and ERK1/2 was involved in luteolin-mediated inhibition of uropathogenic Escherichia coli (UPEC) invasion, and luteolin pretreatment of cells could significantly inhibit the activation of p38 MAPK and ERK1/2.^[81]^ In addition, there are many other non-anti-cancer effects exerted by Luteolin through the regulation of the MAPK pathway, so it is clear that the regulation of the MAPK pathway by Luteolin to exert biological effects is also an important research direction for the future.

### 4.3. Luteolin modulate the NF-κB signaling pathway to exert anti-cancer effects

Nuclear Factor kappa-light-chain-enhancer of activated B cells (NF-κB) is a nuclear transcription factor involved in the transcriptional regulation of the immunoglobulin κ light chain and has functions in the regulation of the immune response and in relation to inflammation. Normally, NF-κB binds to the nucleus as a dimer with κB inhibitors and has no biological activity.^[82]^ The main members of the NF-κB family are: p50 (NF-κB1), p52 (NF-κB2), p65 (Rel A), Rel B and c-Rel. NF-κB exists mainly in the form of dimers formed by p50 and p65 and is inactivated when it binds to IκB (an inhibitor of κB) to form a trimer.^[83]^ The NF-κB pathway has been considered as the most typical pro-inflammatory signaling pathway, consisting mainly of NF-κB, IκB, IKK, receptors and proximal adhesion proteins, and this pathway is further classified into classical or non-classical signaling pathways, where the activation of classical signaling pathways is dependent on the NF-κB essential modulator (NEMO), whereas the non-classical signaling pathways are not.^[84]^

In a cervical cancer study by Zhao et al the expression level of p-NF-κB in HeLa cells was reduced after treatment with luteolin, and p-NF-κB expression was further reduced by the addition of an inhibitor, indicating that luteolin could inhibit the NF-κB pathway in HeLa cells, and it was hypothesized that luteolin could inhibit the proliferation and promote apoptosis of HeLa cells by regulating the NF-κB pathway.^[85]^ In addition, in a study on osteosarcoma, Qu et al found that luteolin can inhibit the proliferation, migration and EMT transformation of U2OS osteosarcoma cells and reduce the expression of related proteins in the NF-κB pathway, suggesting that Luteolin play an anti-osteosarcoma role by inhibiting the activation of the NF-κB signaling pathway.^[59]^ The ability of Luteolin to inhibit the activation of the NF-κB pathway to inhibit cell proliferation and metastasis and to promote apoptosis has also been demonstrated in human tongue squamous cell carcinoma cells.^[61]^ In addition, the above suggests that Luteolin can exert anti-lung cancer effects by regulating the PI3K/AKT and MAPK pathways, and in fact, Luteolin can also promote apoptosis of lung cancer cells by inhibiting the NF-κB pathway, Ju et al^[86]^ found that Luteolin can lead to the accumulation of ROS, which can significantly inhibit the activity of NF-κB and thus increase the susceptibility of lung cancer cells to tumor necrosis factor (TNF)-induced apoptosis. In a study by Yang et al, luteolin was found to upregulate the expression of miR-6809-5p to inhibit the growth of hepatocellular carcinoma cells. The specific mechanism is that this miRNA can target the lipid raft marker protein (FLOT1) in hepatocellular carcinoma cells and reduce its expression to play the role of inhibiting the growth activity of hepatocellular carcinoma cells, and it is worth mentioning that in this study, the overexpression of miR-6809-5p or the downregulation of FLOT1 were both resulted in the inactivation of various signaling pathways such as NF-κB, ERK1/2, and JNK. The above findings suggest that luteolin may lead to the downregulation of FLOT1 expression through the upregulation of miR-6809-5p, and that both of these expression changes may cause the inactivation of pathways such as NF-κB, thus exerting the anti-hepatocellular carcinoma cell growth effect.^[87]^ Furthermore, in another study targeting hepatocellular carcinoma cells, Luteolin were found to exert anti-tumor effects by activating protein kinases through the activation of AMF in HepG2 cells, thereby modulating the NF-κB signaling pathway.^[88]^

Since the NF-κB pathway mainly exerts pro-inflammatory effects, the mechanism by which luteolin exert anti-inflammatory effects by inhibiting the NF-κB pathway has been widely studied, for example, Luteolin ameliorate allergic rhinitis in a rat model by modulating the NF-κB pathway,^[89]^ and slow down the progression of osteoarthritis in a rat model by modulating the NF-κB pathway.^[90]^ In addition, Luteolin can inhibit ROS generation by inducing inactivation of the NF-κB pathway, thereby preventing pyroptosis of THP-1 macrophages.^[91]^ As the anti-inflammatory properties of luteolin may be particularly relevant in cancer,^[22]^ Luteolin exerting anticancer effects through modulation of the NF-κB pathway is also one of the most promising research directions for the future.

### 4.4. Luteolin modulate other related signaling pathways to exert anti-cancer effects

The above three pathways are the main signaling pathways for luteolin to exert anticancer effects, in addition, there are other signaling pathways involved in the anticancer effects of luteolin, due to less research, so here we summarize the research progress on the regulation of other signaling pathways by luteolin to exert anticancer effects.

The Notch signaling pathway is a highly conserved signaling pathway that determines cell fate by participating in cell differentiation, proliferation and apoptosis.^[92]^ In Sun et al’s^[93]^ study, significant alterations in the expression of tumor-associated miRNAs (up-regulation of miR-34a, miR-181a, miR-139-5p, miR-224 and miR-246, and down-regulation of miR-155) were found in luteolin-treated breast cancer cells, and the study went on to find that the expression of such miRNAs could significantly affect the Notch pathway to exert anti-tumor effects, and that alterations in Notch-1 might in turn affect miRNA expression, suggesting that luteolin can modulate the Notch signaling pathway and thus exert anti-tumor effects. In addition, this anti-tumor effect may be associated with a reduction in key factors that promote tumor development such as Notch-1, Hes-1, VEGF, and MMPs. In another study, Notch-1 overexpression induced an increase in VEGF secretion in Hs-746T gastric cancer cells, whereas VEGF secretion was significantly reduced by treatment with luteolin, and the antitumour effects of inhibition of cell proliferation, migration, tubulogenesis in HUVECs and inhibition of angiogenic mimicry (VM) exerted by treatment with luteolin were rescued by Notch-1 overexpression. Moreover, the interaction between gastric cancer cells and HUVECs promotes cell migration in cell co-culture experiments.^[94]^ In the same year, this research team also showed that Luteolin can reverse EMT by inhibiting the Notch signaling pathway, thereby exerting an anti-gastric cancer effect.^[95]^ In another finding, ribosomal S6 kinase (RSK) has been implicated in the development of triple-negative breast cancer, and RSK is able to phosphorylate Y-box binding protein-1 (YB-1) to upregulate the expression of Notch-4, thereby promoting breast cancer growth and drug resistance. As a promising RSK inhibitor, luteolin can significantly inhibit RSK-mediated phosphorylation of YB-1, thereby inhibiting the Notch pathway to exert anti-breast cancer effects.^[96]^

The Wnt/β-catenin signaling pathway is also a highly conserved signaling pathway that regulates cell proliferation and cell behavior in embryos and adults, and dysregulation of the Wnt/β-catenin signaling pathway has been associated with a wide range of pathological conditions and plays a key role in the process of carcinogenesis.^[97]^ In a study by Han et al,^[98]^ it was found that frizzled homologue 6 (FZD6), as a negative regulator of β-catenin transcriptional activity, was able to abrogate prostate cancer cell stemness, while Luteolin were able to upregulate the expression of FZD6, which in turn indirectly negatively modulated the Wnt signaling pathway to exert an inhibitory effect on prostate cancer cell stemness. Also in another study on triple-negative breast cancer, Luteolin were able to inhibit the expression of β-catenin protein and mRNA in vitro and in vivo, thereby reversing EMT and exerting an inhibitory effect on breast cancer metastasis.^[99]^ In addition, Luteolin can reduce azoxymethane (AOM)-induced cell proliferation through the inhibition of GSK-3β, β-catenin, and cyclin D1, thus exerting an anticolon cancer effect.^[100]^

The tyrosine kinase/signal transducer and activator of transcription (JAK/STAT) signaling pathway is involved in many important cellular processes and induces the expression of several key mediators of cancer and inflammation. Dysregulation of this pathway is significantly associated with various cancers and autoimmune diseases.^[101]^ Type I interferon (IFN-α/β) is a multifunctional cytokine with immunomodulatory and anti-proliferative activity that acts by binding to cell surface IFN receptors (IFNAR1/2), activating JAK and subsequently phosphorylating STAT1 and STAT2 proteins, thereby activating the JAK/STAT pathway. In contrast, cyclic AMP-activated protein kinase A (PKA) negatively regulates the IFN-β-induced JAK/STAT pathway. Luteolin reduced intracellular cyclic adenosine monophosphate (cAMP) levels by increasing the cAMP-degrading activity of IFNAR2-bound phosphodiesterase, and the reduction in cAMP levels led to the inactivation of PKA. This suggests that cAMP-activated PKA inhibits the JAK/STAT pathway, whereas luteolin may act on phosphodiesterase to reduce the levels of both cAMP and PKA, thereby enhancing the activation of the JAK/STAT pathway, which in turn sensitizes IFN-α/β to the antiproliferative effects of various cancer cells.^[102]^ In addition, it has been reported that luteolin significantly reduced the phosphorylation of STAT3 in human pancreatic cancer cells PANC-1 and SW1990, thereby inhibiting the activation of the JAK/STAT pathway and ultimately reducing the secretion of EMT and MMP to exert anti-tumor effects.^[103]^ The synergistic effect of luteolin with the chemotherapeutic drug paclitaxel has been confirmed by studies, and in a breast cancer study by Yang et al^[104]^ found that the combination of luteolin and paclitaxel could enhance the expression of TNF Receptor Superfamily Member 6 (Fas), and the enhancement of Fas expression was due to the fact that the combination of the drugs could reduce the phosphorylation of STAT3 in breast cancer cells, suggesting that the combination of luteolin and paclitaxel could inhibit the activation of the JAK/STAT pathway to better exert the anticancer effect. S100 calcium-binding protein A7 (S100A7), as a member of the S100 gene family, has the ability to promote the metastasis of cancer cells,^[105]^ Fan et al^[106]^ found that activation of STAT3 was able to upregulate the expression of S100A7 to enhance the metastatic ability of A431-III cells, whereas luteolin acid was able to inhibit the signaling of STAT3, thereby downregulating the expression of S100A7 and thus exerting anticancer effects. Similarly, studies have reported that claudin-2 is highly expressed in human lung adenocarcinoma tissues and that claudin-2 knockdown reduces cell proliferation, whereas phosphorylated STAT3 up-regulates claudin-2 in A549 cells; however, luteolin reduces the level of STAT3 phosphorylation, thereby down-regulating claudin-2 expression to exert anti-tumor effects.^[107]^ Furthermore, in Fu et al^[108]^ it was found that heat shock protein (Hsp90) overexpression rescued luteolin-mediated degradation of tyrosine (705)-phosphorylated STAT3, and that luteolin also reduced the levels of other proteins that interacted with Hsp90. Then through Co-Immunoprecipitation and Western Blotting, Luteolin were found to induce the degradation of tyrosine (705) and serine (727) phosphorylated STAT3 through a proteasome-dependent pathway, thus blocking the binding of Hsp90 to STAT3, and it was also confirmed that luteolin have a higher affinity for the ATP-binding pocket of Hsp90 in this study. The above suggests that luteolin, by interacting with Hsp90, promote the degradation of Tyr(705)- and Ser(727)-phosphorylated STAT3, which in turn exerts the anticancer effect of inducing apoptosis in cancer cells. It is worth mentioning that similar results to those described above were found in a study on gastric cancer, with the difference that this study also showed that luteolin activate protein tyrosine phosphatase (SHP-1), causing a decrease in the level of STAT3 phosphorylation and a decrease in the expression of related target genes (Mcl-1, Bcl-xl, Survivin).^[109]^

In conclusion, Luteolin can regulate the PI3K/AKT pathway, MAPK pathway, NF-κB pathway, Notch pathway, Wnt/β-catenin pathway, and JAK/STAT pathway to exert anticancer effects, and the specific mechanisms have been described above. In addition, the TGF/SMAD signaling pathway and the Hedgehog signaling pathway may also be the other pathways through which luteolin exert their anticancer effects, but more research is needed in this area, and exploring the anticancer effects of Luteolin through modulation of other pathways will be one of the directions of future research.

## 5. Future directions of luteolin research

The future research direction of luteolin is full of potentials, we found that luteolin play anti-cancer and anti-inflammatory effects of the mechanism is very rich, so we can continue to explore the specific mechanism of luteolin in the cell and physiological processes, we also found that luteolin regulate the relevant pathways often with the help of the intermediate “bridge,” and this “bridge” is usually a certain protein molecule or miRNA. We also found that luteolin often regulate the relevant pathways by acting as a “bridge,” and this “bridge” is usually a specific protein molecule or miRNA. In a study of luteolin inducing the expression of the CYP1A1 and UGT1A1 genes, it was shown that luteolin induce the expression of UGT1A1 and inhibit the expression of CYP1A1.^[110]^ In addition, luteolin have been found to decrease the expression of inducible nitric oxide synthase (iNOS), IL-6, TNF-α, p-NFκBIA (p-IκB-α), p-NFκB p65, and MMP9, while upregulating the expression of Arg-1 and IL-10.^[28]^ This suggests that luteolin may influence the high or low expression of specific genes or enzymes. Therefore, we can continue to explore other new molecules to synergise with luteolin to regulate signaling pathways to better exert anti-inflammatory or anti-cancer effects. In addition, luteolin can synergise with some drugs to exert more effective effects,^[39–41]^ so it is necessary to explore more drugs that can synergise with luteolin. It is worth noting that immunotherapy has been a hot spot in the treatment of tumors in recent years, and in-depth exploration of the ability of luteolin to regulate immune function to exert anti-cancer effects may also be one of the important directions for future research. In addition, the emergence of materials science in recent years has led to the prospect of encapsulating luteolin in nanoparticles to improve bioavailability and efficacy. In the next section, we will focus on the future research direction of luteolin from the aspects of luteolin regulating immune function to play an anticancer role and the research progress of lignan preparations.

### 5.1. Luteolin regulate immune function to exert anti-cancer effects

The immune function mainly includes the body’s immune recognition, immune regulation, immune defence, and the immune function consists of cellular immunity and humoral immunity, and the strength of the immune ability determines the body’s resistance to bacteria, viruses, fungi, mycoplasma, and so on, and at the same time, the dysfunction of the immune function is also significantly related to the development of tumors.^[111]^ The immune function is regulated by various aspects, and some studies have shown that luteolin can regulate the immune function and thus exert anti-tumor effects.

Programmed death receptor 1/programmed death ligand 1 immunosuppressants are currently high-profile immunotherapies. Luteolin significantly inhibits KRAS-mutant proliferative lung cancer and downregulates IFN-γ-induced programmed death ligand 1 expression, acting as an immune checkpoint inhibitor, and there is coordinated potentiation of programmed death receptor 1 inhibitors in combination with luteolin.^[112]^ In a study by Le et al,^[113]^ Luteolin were found to induce activation of APCs through activation of the PI3K/AKT pathway in antigen presenting cells (APCs), enhance cytotoxic T lymphocyte (CTL) responses, inhibit tolerogenic T cells, and exert modulation of immune responses and thus anti-tumor effects.

There are fewer studies on luteolin regulating immune function to exert anti-tumor effects and more studies on luteolin exerting anti-inflammatory and anti-allergic effects by regulating immune function, such as luteolin inhibiting mast cell-mediated allergic inflammation^[114]^ and luteolin exerting neuroprotective effects by inhibiting immune cell activation.^[115]^ Other natural analogues modulating immune function and thus exerting anticancer effects have also been frequently reported, for example, hesperidin (HES) can exert antitumour effects in melanoma cells by modulating immune function in the same way as luteolin.^[116]^ Thus, exploring whether luteolin can be combined with other immune checkpoint inhibitors to exert better anti-tumor effects, exploring whether luteolin can modulate other immune cells to inhibit tumor growth and development, and gaining a deeper understanding of the anti-cancer effects of luteolin in modulating immune functions are the key directions for future research.

### 5.2. Luteolin preparations

Drug delivery systems are solid, liquid or gaseous drug formulations consisting of particles of a specific particle size (micron or nanometer) made from the drug or a suitable carrier by dispersion embedding technology, which has the effect of improving the taste of the drug, increasing the solubility of poorly soluble drugs, increasing the bioavailability of the drug, improving the stability of the drug, reducing adverse effects, delaying the release of the drug and increasing the targeting of the drug.^[117]^ The luteolin drug delivery systems can be divided into liposomes and nanoparticles (NPs), depending on the type of drug.

Liposomes are microscopic vesicles formed by encapsulating a drug in a lipid-like bilayer and are highly biocompatible in vivo. Some investigators prepared liposomes (lipo-luteolin) with a luteolin-cholesterol-soft phospholipid mass ratio of 1:2:7,^[118]^ and the liposomes were slowly released within 120 hours. And the pharmacokinetics suggested that the plasma luteolin concentration of lipo-luteolin was 10-fold higher than that of free luteolin at 2 hours after intravenous injection, and the AUC0~t significantly increased, and the liposome encapsulation significantly improved the bioavailability. In addition, these lipoLuteolin could directly induce apoptosis of tumor cells, reduce tumor angiogenesis and inhibit tumor proliferation.

Mahin et al exploited the hydrophobicity of luteolin to formulate water-soluble copolymer-encapsulated luteolin nanoparticles, followed by the addition of copolymer-encapsulated luteolin nanoparticles to breast cancer cells, which was found to significantly reduce cell viability, increase the production of ROS and calcium ions, and induce cell apoptosis, suggesting that the prepared nanoparticles could be used as potential therapeutic agents for breast cancer.^[119]^ In another study, Fu et al^[120]^ chose the selected [poly(propylene sulfide)-poly(ethyleneglycol) (PPS-PEG)] as a nanocarrier to construct luteolin-loaded nanoparticles (luteolin-PPS-NPs), Luteolin-PPS-NPs has strong hydrophilicity, which is a good solution to the problem of poor water solubility of luteolin. Next, the study found that luteolin-PPS-NPs could kill melanoma cells at low concentrations, resist melanoma cell proliferation, migration and invasion, and promote cancer cell apoptosis. It is worth mentioning that intratumoural administration of luteolin-PPS-NPs showed stronger antimelanoma activity compared to free LUT in animal experiments. This suggests that the prepared nanoparticles can exert anti-tumor effects more efficiently.

In addition to the above 2 drug delivery systems, polymeric micelles, microemulsions and nanoparticles are also included. Nanoparticles are further classified into polymer nanoparticles, protein nanoparticles, lipid nanoparticles, selenium complex nanoparticles and cellulose complex nanoparticles depending on the drug carrier.^[121]^ Based on the above evidence, luteolin has clear potential for the treatment of cancer and the drug delivery system may enhance its anticancer efficacy and improve the hydrophilicity of luteolin while reducing the associated adverse effects. Although the drug delivery system of luteolin has been studied, more systematic studies and clinical validation are still lacking. Future work should focus on the transport, biodistribution and degradation of luteolin formulations, as well as the immune responses and systemic toxicities induced, in order to improve the clinical use of luteolin. In addition, there is evidence that luteolin can synergise with certain molecules or drugs to exert antitumour effects, so whether luteolin and molecules or drugs can target cancer foci through co-delivery systems to exert more effective anticancer effects is also one of the key directions for future research.

## 6. Pharmacokinetics of luteolin

Pharmacokinetics is of paramount importance in the study of new drugs and in the discovery of drug toxicity, and therefore a discussion of the pharmacokinetics of luteolin is of value in guiding future clinical trials. In a study by Chen et al, luteolin was found to be rapidly absorbed when 200 mg/kg of white chrysanthemum extract was administered orally, and plasma luteolin levels peaked 66 minutes after administration.^[122]^ In another study, rats were given luteolin-rich liquorice extract by gavage at a dose of 1 g/kg and the plasmakinetic parameters measured by HPLC-MS/MS were: t_1/2_ was 31.08 ± 1.17 hours, C_max_ was 185 ± 0.12 ng/mL, T_max_ was 0.87 ± 0.05 hours, MRT _(0-t)_ was 20.74 ± 1.91 hours, AUC _(0-t)_ was 35.01 ± 0.81 hng/mL.^[123]^ In contrast, after gavage of rats with luteolin-rich chrysanthemum extract at a dose of 10 g/kg, the parameters measured by the same HPLC-MS/MS technique were: a T_max_ of 0.5 ± 0. 001 hours, a C_max_ of 2079.55 ± 307.09 ng/mL, a T_1/2_ of 8.89 ± 4.01 hours, an AUC _(0-t)_ of 24237.94 ± 2113.55 hng/mL and an AUC_(0-∞)_ of 25094.35 ± 2232.12 hng/mL.^[124]^ Interestingly, significant differences in the pharmacokinetics of luteolin administered alone and in combination with resveratrol were found in the study by Wu et al Co-administration resulted in a 4.8-fold increase in the Cmax of luteolin by the specific mechanism that resveratrol increases the bioavailability of luteolin by decreasing the major metabolite of glucuronide in rats.^[125]^ In another study, pitted olive extract containing 183 ± 5.81 μg of luteolin was administered to rats by gavage and the plasma lignan concentration was 0.01 ± 0.003 μg/mL as measured by LC-ESI-MS/MS 30 minutes later.^[126]^

Luteolin is a compound with complex pharmacokinetic properties in rats, its oral bioavailability is low and it has faster absorption and distribution in rats, with a faster onset of action. Due to its 4 phenolic hydroxyl groups, it is slightly acidic, mostly free in the gastric acid environment, and easily absorbed into the blood by transmembrane transport, and intestinal absorption of weak acids is also faster. The elimination half-life of luteolin is 7.107 hour, indicating that luteolin is eliminated at a moderate rate in rats.^[127]^ It was found that the in vivo blood concentration of luteolin in rats was low after administration of luteolin and the concentration increased significantly after enzymatic hydrolysis, indicating that luteolin exists in the body mainly in the form of glucuronide bond and other forms. The study also found that after oral administration of luteolin to rats, plasma samples were hydrolyzed by enzyme after drug – time curve in the blood concentration at 0.25 and 8 hours there is a peak of absorption, while the absorption of intraperitoneal administration of the drug did not see a double peak, and in the bile excretion of the study found that the luteolin in the bile is mainly in the form of bound form, the concentration of the drug after hydrolysis of bile samples is high in the 1 to 2 hours and 8 to 12 hours, and did not decrease significantly over time, suggesting that the cause of absorption of luteolin is mainly in the form of glucuronide binding. This suggests that the cause of the bimodal absorption is not due to the hepatic-intestinal circulation, and it is speculated that continued bile excretion is one of the reasons for the low blood concentrations after administration.^[128]^ The low bioavailability of luteolin was also confirmed in another study, possibly due to a significant first-pass effect, where after a single 50 mg/kg dose of luteolin was administered as an intravenous push to rats, the half-life of free (unconjugated) luteolin after intravenous administration was shown to be 8.94 hours and conjugated luteolin was shown to be 4.98 hours by non-atrial chamber analysis. The plasma concentration of luteolin peaked at 5.5 μg/mL after 5 minutes and decreased to below the LOQ (100 ng/mL) after 1 hour. At the same dose, the plasma concentration of luteolin was found to have a biphasic distribution over time, divided into a distribution phase and a slow elimination phase, after oral and intravenous administration. This study concluded that luteolin has a large volume of distribution and high clearance, with a bimodal peak after intravenous and oral administration, indicating the presence of enterohepatic circulation.^[129]^ In a recent study, Tu et al found that the absolute oral bioavailability of luteolin in rats ranged from 3.87% to 5.57% in the range of 10 to 120 mg/kg and that the oral absorption of this ingredient may not increase with increasing dose due to the influence of factors such as solubility, membrane permeability and transporter (non-linear metabolism), which means that it will be a challenge to improve the dosage form of the relevant oral formulations.^[130]^ By reviewing the relevant literature,^[131–133]^ it was found that the oral dose of luteolin was mainly concentrated in the range of 10 to 120 mg/kg, with which AUC_(0-∞)_ had a good linear relationship, whereas C_max_ had a good linear relationship only with the dose range of 10 to 80 mg/kg. Therefore, the linear metabolic characteristics of luteolin can be used to better evaluate the oral absorption of the improved dosage form when the dose of luteolin is controlled within 80 mg/kg, which may provide an important basis for the development of the related formulations mentioned above. It is worth noting that the most abundant metabolite of luteolin in rats is known to be luteolin-3-glucuronide, which is responsible for the binding of luteolin and glycosides to glucuronide via phase II enzymes in the small intestine, liver and kidney. The major metabolite of luteolin in human plasma is then luteolin-3-organosulphate due to transfer of phenolsulphonyltransferase in the intestine.^[134,135]^

In conclusion, the study of luteolin pharmacokinetics not only plays an important role in improving bioavailability in the future, but also provides a theoretical basis for the manufacture of future biologics and clinical trials.

## 7. Clinical perspectives and discussion

As the current means of tumor treatment are limited to surgical resection, radiotherapy, chemotherapy, targeted and immunotherapy, postoperative complications and side effects of radiotherapy and drugs often make the treatment effect unsatisfactory for some cancer patients. The use of biologically active components of traditional Chinese medicine, which can specifically target abnormal genes and signaling pathways in malignant tumors to exert anticancer effects, is a hotspot for the future development of anticancer drugs that are more effective, with fewer side effects and higher safety. As a naturally occurring flavonoid with high safety, low side effects, anti-inflammatory and anticancer activities, luteolin have great potential to be developed into novel anticancer drugs for clinical applications. In this paper, we review the mechanism and pharmacokinetics of luteolin in regulating some signaling pathways to exert anti-tumor effects, in order to provide theoretical references for the clinical application of luteolin. At present, the studies on the anticancer effect of luteolin are stuck in the cell and animal stages (Table 1), lacking high clinical basis, the ultimate purpose of theoretical research should support the clinical transformation, and the clinical use of drugs must follow the “evidence-based medicine.” Therefore, based on cell experiments and animal model experiments, large-scale randomized controlled trials (RCTs) are needed to confirm the anticancer effect of luteolin and to explore the optimal dosage of luteolin to exert its anticancer effect, in order to provide a more practical basis for the use of luteolin in cancer therapy and to improve the bioavailability and therapeutic effect of luteolin to a greater extent. Although there are currently no marketed products to support the clinical anticancer effects of luteolin, a search of the ClinicalTrails.gov website (https://clinicaltrials.gov/) for the keyword “luteolin” reveals that there are already clinical trials of luteolin registered. These clinical trials will greatly facilitate the clinical translation of luteolin, which also indicates that luteolin has excellent clinical translation prospects.

**Table 1:** Mechanism of action and dosage of leteolin exerted in different cancers

Type of cancer	Models (cell line/animal)	Dose	Mechanisms
Liver cancer	HepG2/Hep3B	1–10 μM	Regulate autophagy^[136]^
	Huh7/HepG2 BALB/c mice	1–10 μM 50 mg/kg	Arrest cell cycle^[87]^
Lung cancer	A549/H460 BALB/c nude mice	2.5–80 μM 50 mg/kg	Arrest cell cycle^[137]^
	BEAS-2B/H358/H460/H2122/A549 Nude mice/C57BL/6J mice	10-50 Μm 30 mg/kg	Immunoregulation^[112]^
	A549	20–40 μM	Restrain metastasis and invasion^[21]^
	H460	12.5–200 μM	Restrain metastasis and invasion^[138]^
	NSCLC-VECs	5–150 μM	Restrain metastasis and invasion^[32]^
Ovarian cancer	ES2/A2780	30 μM	Inhibit proliferation^[139]^
	Caov-3 cells/HEK293T cells BALB/c nude mice	30 μM 100 mg/kg	Epigenetic modification^[140]^
Colon cancer	HCT116	10–20 μM	Regulate autophagy^[141]^
	SW620	1–10 μM	Regulate autophagy^[142]^
	SW480/SW620	10–60 μM	Immunoregulation^[143]^
	HT29/SNU407	5–80 μM	Spur apoptosis^[144]^
	HT29	10–160 μM	Spur apoptosis^[145]^
	HT29/SW480/SW620/Lovo BALB/c nude mice	10–100 μM 100 mg/kg	Restrain metastasis and invasion^[25]^
	HT29 BALB/C nude mice	50–150 μM 50 mg/kg	Target ferroptosis and pyroptosis^[146]^
Breast cancer	MDA-MB-231	5–20 μM	Inhibit proliferation^[147]^
	MDA-MB-231/BT-20	10–30 μM	Inhibit proliferation^[148]^
	MDA-MB-231	5–40 μM	Restrain metastasis and invasion^[149]^
	MDA-MB-231	1–256 μM	Arrest cell cycle^[150]^
	HCT116/MDA-MB-231	10–50 μM	Immunoregulation^[151]^
	MDA-MB-231 cells/MCF-7	2–50 μM	Regulate metabolism^[152]^
	MCF-7/4T1 BALB/c nude mice	10–100 μM 100 mg/kg	Regulate metabolism^[153]^
	MDA-MB-231	2 μM	Target cancer stem cells^[154]^
	MDA-MB-231/MCF-7 BALB/c nude mice	25, 50, 100 μM 15, 20 mg/kg	Arrest cell cycle^[93]^
	MDA-MB-231	0–15 μM	Spur apoptosis^[104]^
Esophageal cancer	TE-1 BALB/c nude mice	10–40 Μm 50 mg/kg	Target cancer stem cells^[155]^
	Eca109	40–240 μM	Spur apoptosis^[156]^
Malignant melanoma	A375 BALB/c nude mice	5–30 μM 100 mg/kg	Spur apoptosis^[37]^
	SK-Mel2/A375/SK-Mel28 Nude mice	0.25–256 μM 15, 50 mg/kg	Immunoregulation^[157]^
	DC2.4/RAW 264.7/B16F10 C57BL/6 mice	40 μg/mL 30 mg/kg	Immunoregulation^[113]^
Cervical carcinoma	Hela cells	5–20 μM	Epigenetic modification^[158]^
Pancreatic cancer	MIAPaCa2/PANC1	100 μM	Inhibit proliferation^[159]^
Kidney cancer	786-O/OS-RC-2 cells/HK-2 cells BALB/c nude mice	50–100 μM 50 mg/kg	Target ferroptosis and pyroptosis^[160]^
Human Glioblastoma	A172/U-373MG	10–200 μM	Regulate autophagy^[161]^
Human choroidal melanoma	C918/OCM-1	0.1–400 μM	Restrain metastasis and invasion^[162]^
	C918/OCM-1	5–15 μM	Arrest cell cycle^[163]^

μM represents the concentration of luteolin used in cells and mg/kg represents the concentration of luteolin used in in vivo experiments.

However, due to the low bioavailability and poor solubility of luteolin, systemic administration of luteolin may not result in the desired therapeutic effect in clinical trials. Therefore, the focus of large-scale clinical trials should be on improving the bioavailability of luteolin to maximize its anticancer efficacy. For example, drug delivery systems using materials such as nanolipid particles or hydrogels as carriers or structural modifications of luteolin could be used to alter its limitations. In conclusion, research on luteolin is expected to play an important role in promoting human health.

## Author contributions

**Conceptualization:** Rui Wang.

**Investigation:** Rui Wang, Xia Li, Yanhan Xu, Yangyang Li, Weisong Zhang, Rongqi Guo.

**Funding acquisition:** Rui Wang, Xia Li, Jianxiang Song.

**Writing – original draft:** Rui Wang.

**Writing – review & editing:** Jianxiang Song, Xia Li.
